# DNA methylation as an epigenetic mechanism in the regulation of LEDGF expression and biological response in aging and oxidative stress

**DOI:** 10.1038/s41420-024-02076-2

**Published:** 2024-06-22

**Authors:** Biju Bhargavan, Bhavana Chhunchha, Eri Kubo, Dhirendra P. Singh

**Affiliations:** 1 Ophthalmology and Visual Sciences, University of Nebraska Medical Center, Omaha, NE USA; 2 Department of Ophthalmology, Kanazawa Medical University, Ishikawa, 9200293 Japan

**Keywords:** DNA methylation, Methylation, Gene expression

## Abstract

The physiological quantum of stress-inducible transcriptional protein, Lens Epithelium-Derived Growth Factor (LEDGF), is vital for the maintenance of cellular physiology. Erratic epigenetic reprogramming in response to oxidative stress or with advancing age is found to be a major cause in the gene silencing, leading to pathobiologies. Using aging human (h) eye lens/lens epithelial cells (LECs) coupled with redox-active Peroxiredoxin 6 (*Prdx6*)-deficient (*Prdx6*^*−/−*^) mLECs as model systems, herein, we showed that in aging/oxidative stress, the human LEDGF gene was regulated by unique methylation patterns of CGs nucleotides within and around the Sp1 binding site(s) of CpG island of the LEDGF promoter (−170 to −27nts). The process caused the repression of LEDGF and its target, Hsp27, resulting in reactive oxygen species (ROS) amplification and cellular insults. This phenomenon was opposed to the unmethylated promoter in LECs. Clinically, we observed that the loss of LEDGF in the *Prdx6*^*−/−*^ mLECs or aging lenses/LECs, correlating with increased expression of DNMT1, DNMT3a, and DNMT3b along with the methyl CpG binding protein 2 (MeCP2). Upon oxidative stress, the expression of these molecules was increased with the dramatic reduction in LEDGF expression. While demethylating agent, 5-Aza deoxycytidine (5-AzaC) transposed the aberrant methylation status, and revived LEDGF and Hsp27 expression. Mechanistically, the chloramphenicol acetyltransferase (CAT) reporter gene driven by the LEDGF promoter (−170/ + 35) and ChIP assays uncovered that 5-AzaC acted on GC/Sp1 sites to release LEDGF transcription. The data argued, for the first time, that de novo methylation of CGs around and within Sp1 sites of the CpG island directly disrupted Sp1 activity, which ensued in LEDGF repression and its biological functions. The findings should improve our understanding of cellular insults-associated with aberrant DNMTs-mediated LEDGF’s activity, and can offer strategies for therapeutic intervention to halt aging/oxidative stress-induced abnormalities.

## Introduction

Aberrant epigenetic reprogramming-driven signaling in response to oxidative stress or aging is a major miscreant in the silencing of genes, leading to many aging-related pathobiology and disease states [1–7]. The control of gene expression in mammals by methylation of cytosine residues at CpG dinucleotides in the 5′- flanking regions of genes are mostly enriched in CpG nucleotides, and are designated as CpG islands [8]. In normal cells, CpG islands, specifically associated with promoter segments, are mostly present in unmethylated form [9]. Mostly, DNA methylation represses gene transcription directly by interfering with the interaction of transcription factors to GC and indirectly by recruiting methyl-CpG-binding proteins (MeCPs). Nevertheless, DNA methylation can involve interaction(s) between DNA methyltransferases (DNMTs) and one or more DNA-associated molecules [10]. The nuclear protein LEDGF is an ubiquitously expressed stress-inducible gene, and plays diversified roles depending upon its cellular abundance [11–14]. Previously, we have demonstrated that the LEDGF gene is transcriptionally regulated through Sp1 response elements of the promoter, but its expression is repressed in response to UV (ultra violet)-B stress [15]. The proximal promoter region of LEDGF gene contains CpG island [11, 16]. CpG islands are nearly present in all ubiquitous/housekeeping genes, like LEDGF, though the number of genes can be expressed in cells/tissues specific and cellular microenvironment-dependent manner [3].

Aging or oxidative-driven abnormal DNA methylation has been shown to be involved in the development of many age-related pathobiology [5, 17, 18]. Studies have now dictated the role of DNA methylation in the dysregulation of the genes related to aging-pathobiology [6]. Intriguingly, both aging and oxidative stress possess common molecular denominators and are found to be the major culprits for several aging-related diseases [19, 20]. One of the most common features of aging-diseases is the functional loss of the protective genes with advancing age [21, 22]. This functional decline of anti-death factor genes, such as Nrf2 or LEDGF, causes increased reactive oxygen species (ROS)-dependent pathogenic disorders, including cataractogenesis [11, 12, 14–16, 23, 24]. Upon oxidative stress, the aberrant methylation patterns can influence either activation (hypomethylation) or deactivation (hypermethylation) of genes. However, the mechanisms of the cross-talk between oxidative stress and DNA methylation remain obscure, at least in eye lenses/LECs, as DNA methylation is also tissue-specific. It has been revealed that in mammals, the addition of methyl (CH3) radicals to the cytosine nucleotide is catalyzed mainly via three different DNMTs, namely DNMT1, 3a, and 3b. Also, MeCP2 has been shown as a transcription repressor targeting methylated DNA at CpG islands to silence the genes [25, 26].

LEDGF expression is elevated in LECs during a lower prevalence of ROS [15, 27]. While LECs exposed to oxidative inducers for a longer period or at higher doses, result in the repression of the LEDGF mRNA, suggesting that the repression of LEDGF could be at the transcriptional level [11, 15, 28, 29]. Moreover, LEDGF has several well-characterized domains, such as PWWP and integrase binding domain (IBD), which are responsible for chromatin and protein-protein interaction, respectively. LEDGF exerts its cellular function via protein-protein or LEDGF-chromatin/DNA interaction [30–32]. LEDGF has also been found to be involved in the initiation or progression of several life-threatening disorders, ranging from cancer to HIV-chromatin integration [33–36], including autoimmune diseases [37, 38]. Although the features and biological functions of LEDGF have been studied, little is known about how dynamical change(s) in LEDGF expression occurs and how this is regulated in normal as well as within redox cellular microenvironments, including during aging.

In this study, we identified, for the first time that the LEDGF gene is regulated by methylation of GC sequences residing within the CpG island of the promoter. Mechanistically, aberrant methylation of these GC nucleotides during aging/oxidative stress led to LEDGF silencing. Also, recruitment of MeCP2 and HDAC1 occurred to the GC/Sp1 sites of the promoter, blocking the accessibility of Sp1 to its elements. Notably, this adverse event could be reversed by 5-Aza deoxycytidine (5-AzaC) treatment, leading to the revival of LEDGF expression with increased cell viability. Additionally, we found that the loss of LEDGF in aging-related cataractous lenses was linked to methylation of CpG island (Fig. 6), demonstrating the involvement of aberrant epigenetic reprogramming in the onset of disease state. We consider that the identification of the DNA methylation of LEDGF gene marks should be used to uncover early predictors and define therapeutic agents to reverse abnormal epigenetic changes.

## Results

### LEDGF expression progressively decreased during aging and in cataract lenses/LECs

**Fig. 1:**
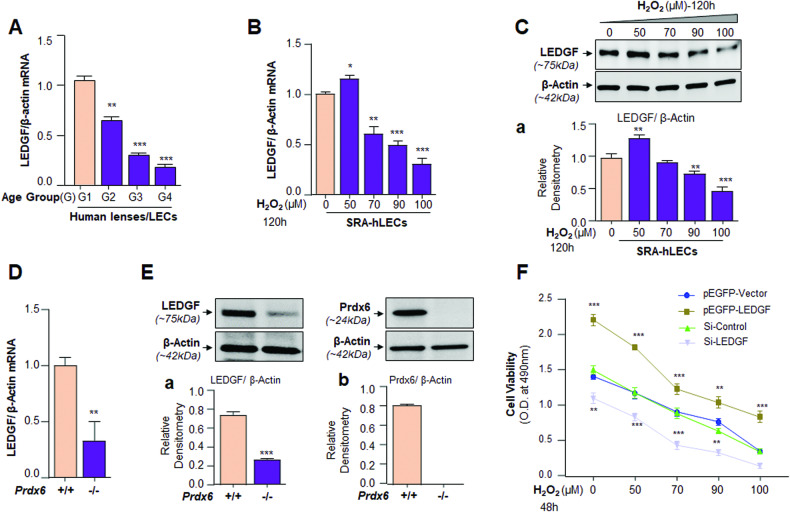
LEDGF expression deteriorated during aging and chronic oxidative stress, and LEDGF expression was required to rescue LECs against oxidative stress. **A** Real-time PCR (qPCR) analysis of LEDGF expression in human lenses of varying age groups. Total RNA was isolated from the lenses of variable ages and processed for qPCR, and results were analyzed and presented as histograms. β-actin was used as a control. Group1 (G1), 18 to 26 y (*n* = 6 lenses); G2, 46 to 52 y (*n* = 6 lenses); G3, 64 to 68 y (*n* = 6 lenses) and G4, 75 to 81 y (*n* = 6 cataractous lenses). Data represent Mean ± S.D. of three independent experiments. G1 vs G2, G3 and G4, **p < 0.001; ***p < 0.0001. A significant reduction in LEDGF mRNA expression was observed with advancing age as shown. **B** Oxidative load-dependent loss of LEDGF mRNA in SRA-hLECs during oxidative stress. qPCR analysis was performed with mRNA isolated from SRA-hLECs after exposures to H_2_O_2_ for 120 h. Histograms represent the Mean ± S.D. of three independent experiments. Control vs H_2_O_2_ treated SRA-hLECs, **p < 0.001; ***p < 0.0001. **C** A decline in levels of LEDGF protein was directly linked to the magnitude of oxidative stress. Cellular extract isolated from SRA-hLECs treated with different concentrations of H_2_O_2_ for 120 h. An equal amount of protein was immunoblotted using an antibody specific to LEDGF as shown. Ca Densitometric quantitation of LEDGF protein bands was normalized with corresponding β-actin protein bands and shown. Histograms represent the Mean ± S.D. of three independent experiments. Control vs H_2_O_2_ treated SRA-hLECs, **p < 0.001; ***p < 0.0001. **D** Redox-active *Prdx6*^*−/−*^ a model for aging, dis*p*layed reduced expression of LEDGF mRNA. Total RNA was isolated from *Prdx6*^*+/+*^ and *Prdx6*^*−/−*^ mLECs, and qPCR was conducted using primers specific to LEDGF. β-actin was used as an internal control. Histograms represent the Mean ± S.D. of three independent experiments. *Prdx6*^*+/+*^ vs *Prdx6*^*−/−*^, **p < 0.001. **E** *Prdx6*^*‒/‒*^mLECs showed reduced expression of LEDGF protein. Cellular extract isolated from *Prdx6*^*‒/‒*^ and *Prdx6*^*+/+*^ mLECs containing an equal amount of protein was immunoblotted using antibodies as indicated in Figure. β-actin served as loading control, Immunoblot Image is a representative of three independent replicates. **E** a and b Densitometric analysis of LEDGF and Prdx6 protein bands was normalized with corresponding β-actin protein bands. Histograms represent the Mean ± S.D. values of three independent experiments. ***p < 0.0001. **F** Loss-or gain-of-function experiments with LEDGF demonstrated that LEDGF was vital for cell survival in response to oxidative stress. Cell viability assay (MTS assay) was performed to examine the cellular viability of SRA-hLECs under or over-expressing LEDGF facing different concentrations of H_2_O_2_-exposure (50, 70, 90, and 100 μM). Transfection efficiency was normalized with GFP O.D. values measured at Ex485/Em530. Values are Mean ± S.D. of three independent experiments. Asterisks indicate a statistically significant difference. pEGFP-Vector vs pEGFP-LEDGF and Si-Control vs Si-LEDGF, **p < 0.001; ***p < 0.0001.

To examine the fate of LEDGF in lenses/LECs of variable ages, we isolated lenses-derived from deceased healthy human subjects of variable age groups (G) and determined the expression of LEDGF. qPCR of RNA isolated from human lenses, ranging from 18 years (y) to 81 years old showed a significant reduction in the LEDGF mRNA expression with advancing age, and maximal suppression was observed in G4, 75–81y-old lenses/LECs (Fig. 1A). These data demonstrated that LEDGF mRNA expression is inversely associated with advancing age and pointed out the deterioration of the transcriptional machinery during aging, at least in lens/LECs. Based upon the increasing studies, including our group [15, 16] showing that DNA methylation can be a causative event in gene silencing, we postulated that LEDGF gene containing CpG island with Sp1 binding sites (GC boxes) may be methylated. This allowed us to design this study to unravel the role of DNA methylation of the LEDGF gene and its impacts on LECs’ health.

### A physiological abundance of LEDGF rescued LECs from oxidative cell damage

To investigate the effect of increased oxidative stress on LEDGF expression in SRA-hLECs. We exposed the SRA-hLECs to different concentrations of H_2_O_2_ for 120 h (Fig. 1B). qPCR and immunoblot analyses showed that H_2_O_2_-induced oxidative stress significantly decreased the expression of LEDGF at 70–100 μM of H_2_O_2_ concentrations (Fig. 1B, C), suggesting that the decrease of the protein is directly linked to the levels of LEDGF transcripts. Next, we examined if similar to SRA-hLECs facing oxidative stress, *Prdx6*^*−/−*^ mLECs, a model for aging, show the repression of LEDGF expression. The results of the experimentation demonstrated that LEDGF expression was downregulated in redox-active *Prdx6*^*−/−*^ mLECs compared to *Prdx6*^*+/+*^ as shown in Fig. 1D, E. Next, we tested if LEDGF abundance affects cellular fate against oxidative stress. Using experiments with the loss-and gain-of-function of the LEDGF gene; we found that SRA-hLECs overexpressing LEDGF (pEGFP-LEDGF) facing oxidative stress showed a significant increased cell survival (Fig. 1F). While knocking down the LEDGF gene dramatically decreased cell viability by 73% compared to control (Fig. 1F). Further, we exposed these transfectants to increasing concentrations of H_2_O_2_. Data revealed that transfectants overexpressing LEDGF conferred resistance and there was an increase of 55% viable cells compared to control vector-transfectants when exposed to H_2_O_2_ at 50 μM concentration (Fig. 1F). Overall, the data demonstrated that LEDGF expression is requisite for LECs survival against oxidative stress.

### Levels of oxidative stress influenced LEDGF transcription

**Fig. 2:**
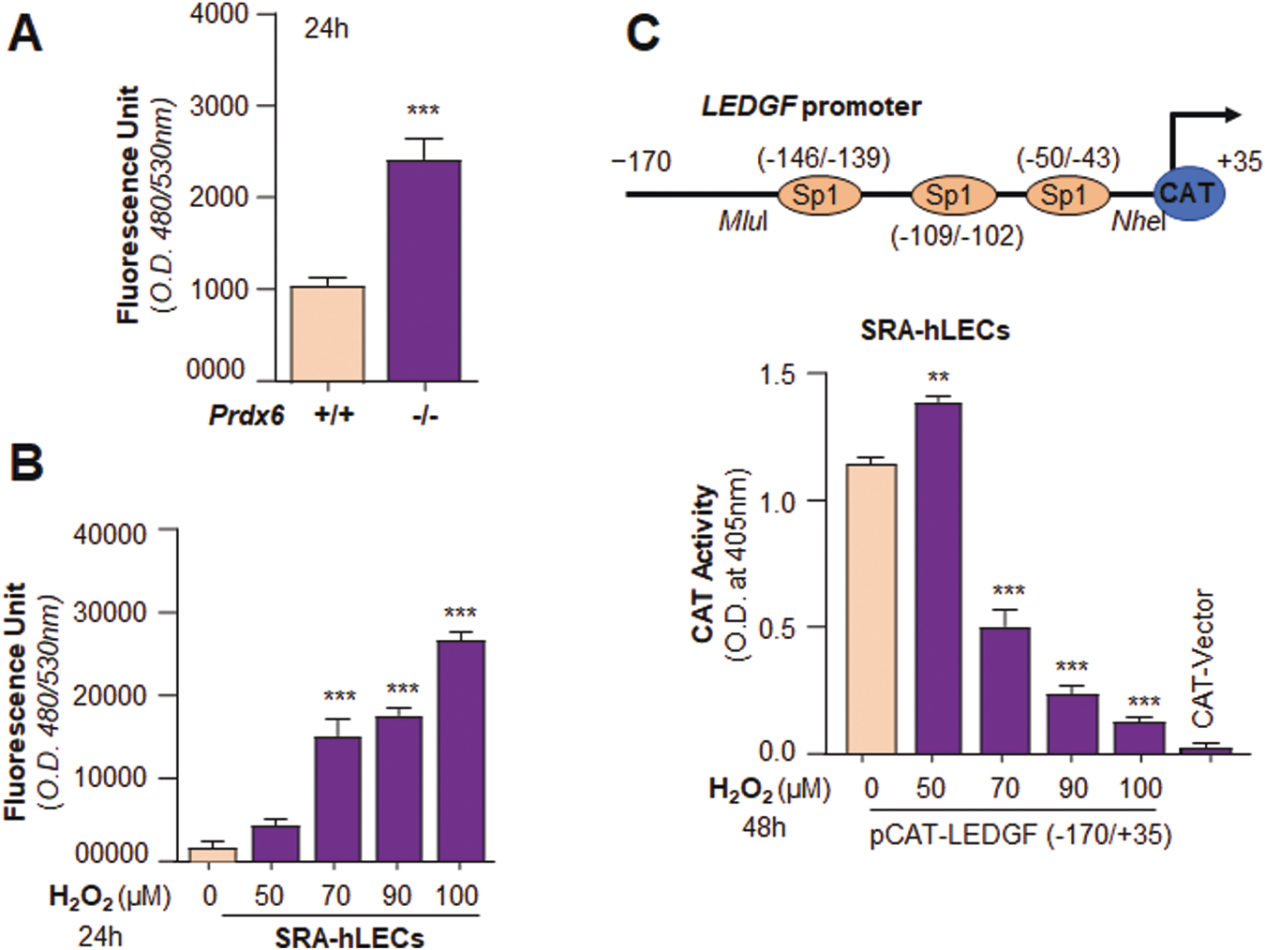
SRA-hLECs under oxidative stress or *Prdx6*^*‒/‒*^-deficient mLECs (a model for aging) displayed increased levels of oxidative load, which was inversely linked to LEDGF transcription. **A** ROS levels of cultured *Prdx6*^*+/+*^ and *Prdx6*^*‒/‒*^ mLECs (an aging model) were assessed by replacing the medium with Hank’s balanced medium containing 10 μM of H_2_-DCF-DA at Ex485/Em530 nm. The data were represented as Histograms. Values are Mean ± S.D. of three independent experiments. *Prdx6*^*+/+*^ vs *Prdx6*^*‒/‒*^; ***p < 0.0001. **B** Intracellular ROS of cultured SRA-hLECs exposed to different concentrations of H_2_O_2_ were measured by H_2_DCF-DA dye as indicated. Histograms represent the Mean ± S.D. of three independent experiments. Control vs H_2_O_2_ treated LECs, ***p < 0.0001. **C** LEDGF promoter assay disclosed that oxidative stress disrupted its transcriptional activity. The promoter activity assay was performed by co-transfecting SRA-hLECs with LEDGF-CAT reporter plasmid containing all Sp1 sites (−170/ + 35 bp) as shown in **C**, upper panel or empty CAT vector along with pEGFP vector. Transfectants were exposed to variable concentrations of H_2_O_2_ to produce oxidative stress as shown. After 48 h lysate was prepared and CAT activity was measured and presented. Transfection efficiencies were normalized with protein concentration as well as GFP values (O.D. at Ex485/Em530). Histograms represent the Mean ± S.D. values of three independent experiments. Asterisks indicate a statistically significant difference between Control vs H_2_O_2_ treated SRA-hLECs, **p < 0.001, ***p < 0.0001.

To examine if the transcription of LEDGF is disrupted under oxidative pressure, we quantified ROS by H_2_DCF-DA dye in *Prdx6*^*+/+*^ and *Prdx6*^*−/−*^ mLECs (a model for aging). As expected, the data demonstrated an increase of ROS levels in *Prdx6*^*−/−*^ mLECs compared to *Prdx6*^*+/+*^ (Fig. 2A). Similar to mLECs, we found an increase in ROS levels in SRA-hLECs was directly connected with the increasing concentrations of H_2_O_2_ treatment (Fig. 2B). These observations indicated that both oxidative stress and aging generate excessive ROS in LECs. We then examined the net effect(s) of oxidative load on LEDGF transcription. SRA-hLECs were transfected with LEDGF promoter (−170/+35nts)-linked to CAT plasmid (pCAT-LEDGF) containing all three Sp1 sites [11], and subjected to different magnitudes of oxidative stress by exposing to increasing concentrations of H_2_O_2_ (Fig. 2C). Data analysis revealed a significant decrease in the promoter activity, which was linked to oxidative stress levels (Fig. 2B). Collectively, our data suggested that the excessive oxidative stress represses the LEDGF transcription.

### Oxidative stress altered the DNMTs expression in LECs

**Fig. 3:**
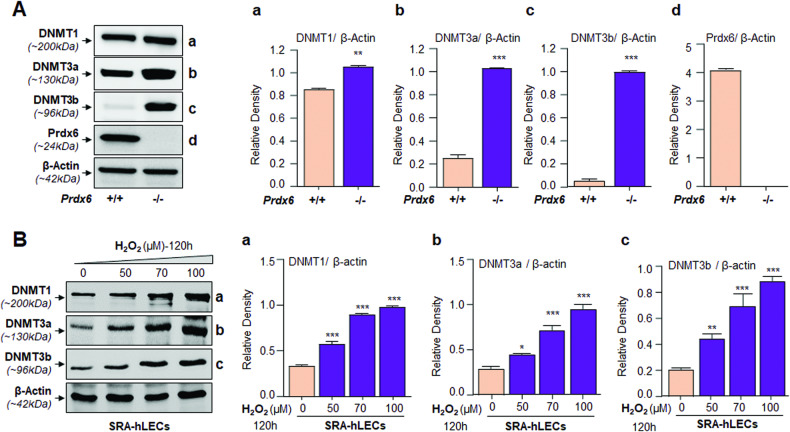
*Prdx6*^*−/−*^-deficient mLECs, a model for aging cells or SRA-hLECs under oxidative stress displayed increased levels of DNMTs. **A** *Prdx6*^*‒/‒*^harboring increased accumulation of ROS showed increased levels of DNMTs. Nuclear extract isolated from *Prdx6*^*+/+*^ and *Prdx6*^*‒/‒*^ mLECs having an equal amount of protein was immunoblotted using antibodies-specific DNMTs and Prdx6 as indicated. β-actin served as internal loading control. Histograms show densitometric analysis of protein bands of DNMT1/β-actin (Aa), DNMT3a/β-actin (Ab), DNMT3b/β-actin (Ac) and Prdx6/β-actin (Ad) Mean ± S.D. of three independent experiments: *Prdx6*^*+/+*^ vs *Prdx6*^*‒/‒*^; **p < 0.001, ***p < 0.0001. **B** SRA-hLECs exposed to increasing concentrations of H_2_O_2_ displayed increasing levels of DNMTs. Cellular extract isolated from SRA-hLECs having equal amounts of protein was immunoblotted using antibodies specific to corresponding DNMTs as shown. The same membranes were used to re-probe with antibodies after membrane restriping. β-actin antibody served as an internal loading control. Histograms represent the Mean ± S.D. values of relative densitometry of protein bands of DNMT1/ β-actin (Ba), DNMT3a/β-actin (Bb), DNMT3b/β-actin (Bc) from three independent experiments. Control vs H_2_O_2_ treated SRA-hLECs samples, *p < 0.01, **p < 0.001, ***p < 0.0001.

Because this process is dependent upon the status of DNMTs enzymes that catalyze(s) the addition of methyl groups to the 5′ carbon of the cytosine residues, we examined the levels of DNMTs in redox active, *Prdx6*^*−/−*^ mLECs or SRA-hLECs under oxidative stress. Cellular extract from *Prdx6*^*−/−*^ and *Prdx6*^+/+^ mLECs and SRA-hLECs facing oxidative stress induced by different concentrations of H_2_O_2_ were immunoblotted with antibody specific to DNMTs or β-actin as internal control (Fig. 3). *Prdx6*^*−/−*^ cells showed elevated levels of DNMTs protein compared to *Prdx6*^*+/+*^ mLECs (Fig. 3A). Similarly, SRA-hLECs exposed to H_2_O_2_ displayed increased expression of DNMT1, DNMT3a, and DNMT3b (Fig. 3B). The data derived from the experiments indicated the plausible involvement of aberrant DNA methylation in LEDGF silencing.

### Methylation of CpG dinucleotides negatively affected the LEDGF promoter activity, and 5-AzaC treatment revived the process

**Fig. 4:**
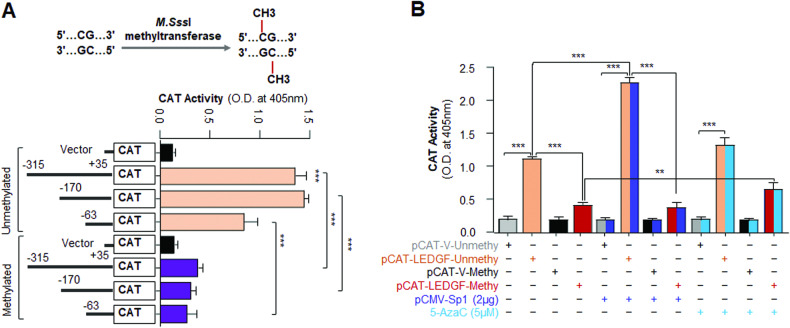
Methylation of CpG dinucleotides present in 5-proximal promoter disrupted LEDGF transcriptional activity. **A** Promoter activity assay was performed by co-transfecting LECs with *M.Sss*I methyltransferase-mediated in-vitro methylated or unmethylated LEDGF-CAT reporter plasmid constructs (−315/ + 35, − 170/ + 35, −63/ + 35 bp) or empty CAT vector along with pEGFP vector for transfection normalization. After 48 h transfectant lysate was prepared and CAT activity was measured as noted in Materials and Methods. Mean ± S.D. values of CAT values derived from three independent experiments were presented in the form of histograms. pCAT-Vector vs pCAT-LEDGF, unmethylated LEDGF-CAT vs methylated LEDGF-CAT; ***p < 0.0001. **B** Extrinsic expression of Sp1 failed to stimulate the methylated promoter of LEDGF, while 5-AzaC treatment released the promoter activity. The promoter activity of LEDGF was performed by co-transfecting SRA-hLECs with methylated or unmethylated LEDGF-CAT reporter plasmid (− 170/ + 35 bp) or empty CAT vector along with pCMV-Sp1 or pEGFP vector for transfection normalization. After 48 h, lysate was prepared and CAT activity was measured as mentioned in Materials and Methods. Transfection efficiencies were normalized with protein concentration as well as GFP values (O.D. at Ex485/Em530). Histograms represent the Mean ± S.D. values of three independent experiments. Asterisks indicate statistically significant differences **p < 0.001, ***p < 0.0001.

Next, we planned to examine whether indeed, DNA-methylation of the LEDGF promoter at the CpG region containing GC/Sp1 sites silences LEDGF transcription, we performed an in-vitro methylation assay using *M.Sss*I methyltransferase enzyme and three deletion constructs containing GC/Sp1 sites (−315/+35nts, −170/+35nts, −63/+35nts). The methylated fragments were ligated into a CAT vector and transfected to SRA-hLECs. Assay of the promoter activity showed a significant reduction in the promoters activity of the methylated promoter in transfectants (Fig. 4A), emphasizing that the methylation of CpG nucleotides (GC/Sp1) repressed Sp1 transcriptional potential. While the unmethylated promoters displayed a significant increase in the activity in transfectants as shown in Fig. 4A. Next, we examined the effect of ectopic expression of Sp1 in regulating methylated and unmethylated LEDGF promoter. We selected the critical region of the promoter, spanning from −170/+35nts, and subjected it to methylation by the M.*Sss*I enzyme. The purified methylated promoter or unmethylated promoter was co-transfected with pCMV-Sp1 in SRA-hLECs and were treated/untreated with 5-AzaC (5 µM), and assessed for the promoter activity. We found that compared to the unmethylated promoter, the methylated promoter showed a dramatic decrease in its activity, and even ectopic expression of Sp1 could not alter the promoter activity of methylated promoter (Fig. 4B). To confirm the results, we employed a demethylating agent, 5-AzaC. We observed that treatment of transfectants with 5-AzaC reversed the process as indicated in Fig. 4B, suggesting that the methylation of critical CpG nucleotides at the LEDGF promoter negatively regulates LEDGF transcription.

### Oxidative stress disrupted the binding of Sp1 by enhancing the interaction of HDAC1, 5-methyl cytosine, and MeCP2 at Sp1 binding sequences

**Fig. 5:**
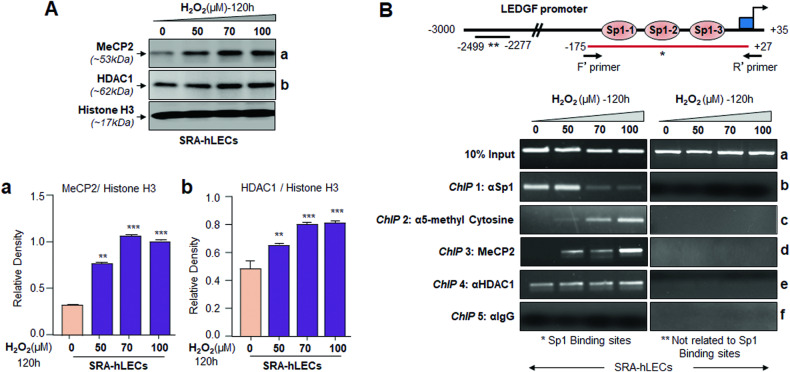
Sp1 binding sites within CpG island of LEDGF promoter were enriched with MeCP2, 5-methyl cytosine, and HDAC1, and the enrichment led to the inhibition of Sp1/GC interaction. **A** Immunoblot analysis showing oxidative load-dependent changes in MeCP2 and HDAC1 expression. Total extract isolated from cells facing different concentrations of H_2_O_2_ containing equal amounts of protein was immunoblotted using specific antibodies to the corresponding molecules as shown. Densitometric analysis of the MeCP2 (Aa) and HDAC1 (Ab) protein band values normalized with corresponding Histone H3 and were presented as histograms. The data represents Mean ± S.D. values from three independent experiments. Control vs H_2_O_2_ treated LECs, **p < 0.001, ***p < 0.0001. **B** ChIP experiments revealed the oxidative load-dependent recruitment of MeCP2, HDAC1, and enrichment of 5-methyl cytosine at CpG island and impaired Sp1/GC binding. Chromatin immunoprecipitation analysis was performed with chromatin extracted from SRA-hLECs exposed to increasing concentrations of H_2_O_2_ using ChIP grade specific antibodies of corresponding molecules as indicated. The top panel shows the LEDGF promoter and the location of PCR primers designed for the assay. DNA fragments present in the immunoprecipitates were amplified with primers that specifically recognize a fragment of the LEDGF promoter containing CpG/Sp1 binding sites (Left panel; *, −175 to +27). As a negative control, primers designed beyond the Sp1 sites (Right panel; **, −2499 to −2277) or a mock ChIP with control IgG (Bf) were used to validate the results. PCR products were separated on 2.5% agarose gels, stained with ethidium bromide, and recorded. Images are representative of three experiments. All three binding sites of Sp1 at the LEDGF promoter were adjacent to each other, therefore, we were unable to make the primers for each site for the experiments. In this context, we would like to mention our previous reports [11, 16], which showed that all three Sp1 sites are functional and differentially regulate LEDGF transcription.

To examine the status of MeCP2 and HDAC1 in response to oxidative stress, cellular extract isolated from SRA-hLECs treated with H_2_O_2_ was immunoblotted with antibodies specific to MeCP2 and HDAC1. Results showed H_2_O_2_ concentration-dependent increase of MeCP2 and HDAC1 expression (Fig. 5A) Together, the data demonstrated that oxidative stress inducts expression of MeCP2 and HDAC1 in SRA-LECs. Further, to examine the interaction of Sp1 as well as enrichment of HDAC1, 5-methyl cytosine and MeCP2 to the Sp1/GC binding sites of LEDGF promoter, we performed chromatin immunoprecipitation (ChIP)-RT-PCR using specific antibodies; As shown in Fig. 5B, the amplification of the Sp1 antibody pulled fragment of the promoter indicated that the enrichment of Sp1 to its sites were significantly reduced due to oxidative stress (5B, b). Next, we examined the interaction of 5-methyl cytosine (Fig. 5B, c), MeCP2 (Fig. 5B, d), and HDAC1 (Fig. 5B, e) at the Sp1 interacting sites. Chromatin pull down with 5-methyl cytosine, HDAC1, and MeCP2 antibodies showed increased enrichment of these proteins at the Sp1/GC sites (Fig. 5B). We did not see any band in the control samples as indicated in figure, pointing specificity of results. The results revealed the involvement of 5-methyl cytosine, MeCP2, and HDAC1 in blocking Sp1 engagement to its sites.

### Sp1 interacting sites of CpG island residues in the LEDGF promoter were methylated in aging non cataractous and cataractous lenses/LECs, and oxidative stress

**Fig. 6:**
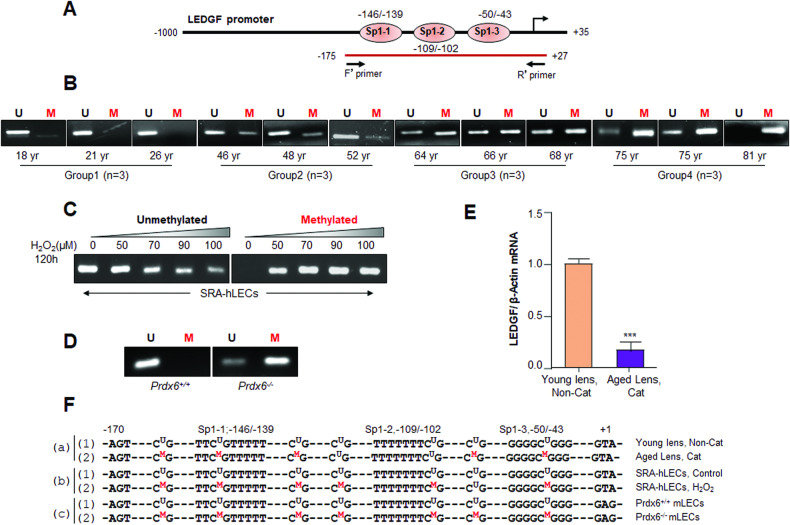
LEDGF promoter was increasingly methylated with increasing of age and oxidative loads. **A** Diagrammatic illustration of the promoter of LEDGF region with Sp1/GC sites with primers positions used for the M.S.P. experiments. **B** Methylation and unmethylation detection experiments demonstrated that the promoter of LEDGF is increasingly methylated with the advancing age of human lenses. As expected, we observed increased methylation levels in cataractous lenses. M.S.P. analysis was performed in human lenses isolated from healthy deceased subjects of various age groups, including the cataractous group (Group 4) as indicated in Figure. Images are representative of three independent experiments. **C** M.S.P. analysis disclosed that LEDGF promoter (−175/ + 27) containing Sp1 sites within the CpG island was methylated in response to oxidative stress. M.S.P. analysis was conducted in LECs after exposure to increasing concentrations of H_2_O_2_ for 120 h as described in Materials and Methods. Images are representative of three independent experiments. **D** A model for aging, redox-active *Prdx6*^*‒/‒*^ mLECs had methylated promoter of LEDGF, while *Prdx6*^*+/+*^ mLECs did not as evidenced by M.S.P. experimentation. M.S.P. analysis was carried out in *Prdx6*^*+/+*^ and *Prdx6*^*‒/‒*^ LECs. Images are representative of three independent experiments. **E** Levels of LEDGF mRNA in cataract lenses showed a dramatic reduction in expression levels, which was linked to increased methylation of LEDGF DNA. Total RNA was isolated from lenses and subjected to qRT-PCR using primers specific to LEDGF. β-actin primers served as internal control. Data represent Mean ± S.D. of three independent experiments. ***p < 0.0001. **F** The bisulfite sequenced nucleotides of M.S.P. analyzed DNA isolated from human lenses of young (a1), cataract (a2) non-cataract, SRA-hLECs untreated (b1), SRA-hLECs exposed to H_2_O_2_ (b2), *Prdx6*^*+/+*^ mLECs (c1) and *Prdx6*^*‒/‒*^ mLECs (c2).

CpGPlot program analysis of LEDGF promoter spotted a CpG island (CGI) spanning from −170 to +1nts as shown in Fig. 6F. CGI stretch is mostly unmethylated CpG sequences and occupies transcription start site (TSS) and can be methylated [39]. Since the CGI of the LEDGF promoter has similar pattern, we postulated that the promoter might be a target for methylation during oxidative stress. Therefore, we performed methylation-specific PCR (MSP) to determine the methylation status of involved GC residues of the promoter in aging hLECs, SRA-hLECs facing oxidative stress and in redox active *Prdx6*^−/−^ mLECs, including human cataractous vs non-cataractous lenses/LECs. The results revealed a significant correlation between aging or cataract and the promoter methylation pattern (Fig. 6B). We identified a progressive increase of methylated promoter bands in aging lens samples; Group 2, Group 3, and Group 4 cataractous lenses when examined (Fig. 6B). We did not detect any methylation in Group 1 lens/LECs samples (Fig. 6B). Interestingly, we observed the increased methylation status of the promoter (Fig. 6B) was directly linked to the increased silencing of LEDGF expression (Fig. 6E).

Because aging lenses/LECs bore the methylated LEDGF promoter and aging hLECs harbored increased levels of oxidative stress [24], we examined the net effect of oxidative pressure-induced changes in the methylation status of the promoter. Genomic DNA isolated from SRA-hLECs facing different magnitudes of oxidative stress were processed for bisulfite conversion sequencing using PCR with the specific region (CpG) of the promoter-specific methylated/unmethylated primers. As shown in Fig. 6, methylation-specific primers could amplify the specific region of the promoter in H_2_O_2_ treated samples (Fig. 6C), demonstrating that the promoter is methylated in SRA-hLECs during oxidative stress. Similarly, we also examined the methylation status of the mouse LEDGF promoter, since this region of human and mice is evolutionary well conserved [11, 16]. As expected, the data analysis revealed that the mouse LEDGF promoter was methylated in *Prdx6*^−/−^ mLECs, but was not in *Prdx6*^*+/+*^ (Fig. 6D).

### Critical CpGs of LEDGF promoter are methylated in aging or oxidative stress and cataractous lenses

Because MSP analysis showed methylation of LEDGF promoter in redox active LECs and cataractous lenses, we mapped the critical CpGs’, which were targets for methylation. We cloned the DNA fragment obtained via MSP analysis into a pCR^TM^4-TOPO^®^ vector. The sequencing of the cloned DNA revealed the presence of the specific methylated CpGs’ in the promoter. We found 12 critical CpG dinucleotides between −170 to +1nts region (Fig. 6F) containing all three Sp1 binding sites. We observed 100% methylation at the Sp1 site-1 and Sp1 site-3 in the aging cataractous lens (75 yr). However, we could not observe any methylation at Sp1 site- 2 (Fig. 6F, a). Additionally, we identified that H_2_O_2_ treatment caused 100% methylation at Sp1 site-1 and site-2, and ~50% at Sp1 site-3 (Fig. 6F, b). However, the LEDGF DNA from *Prdx6*^−/−^ mLECs showed 100% methylation of all Sp1 sites compared to *Prdx6*^+/+^ mLECs (Fig. 6F, c).

### 5-AzaC treatment reversed the oxidative stress-induced promoter methylation and released LEDGF transcription with increased cell viability

**Fig. 7:**
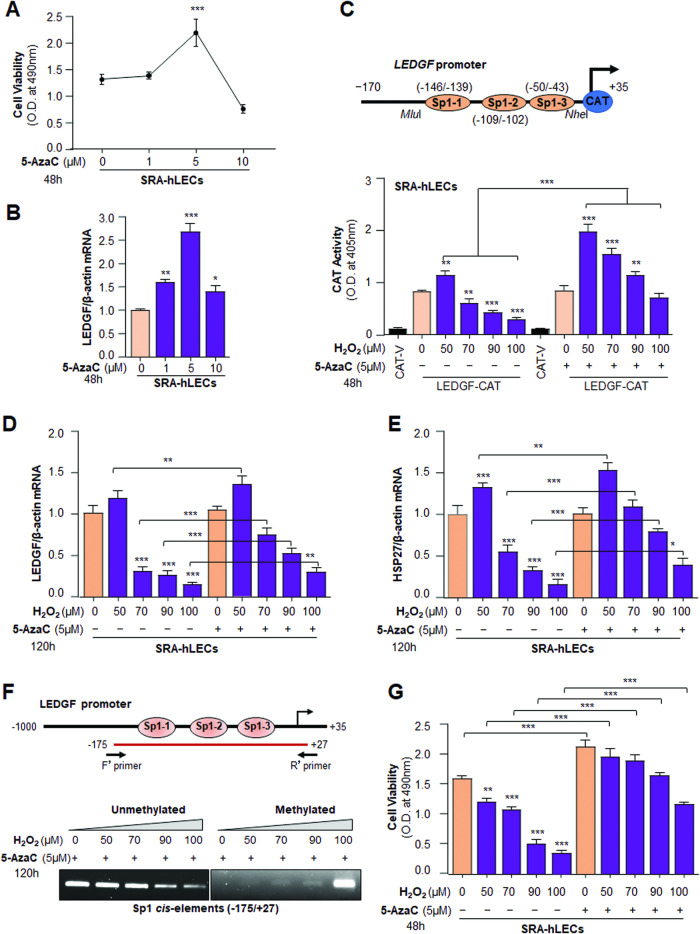
5-AzaC treatment enhanced LECs viability and LEDGF expression. **A** MTS assay was used to assess SRA-hLECs viability after treatment with 5-AzaC for 48 h, suggesting 5 μM was non-toxic and better for SRA-hLECs health as shown. Control vs 5 μM 5-AzaC treatment, ***p < 0.001. **B** 5-AzaC treatment upregulated expression of LEDGF transcript. In a parallel experiment, SRA-hLECs were treated with 5-AzaC. Total RNA was isolated and processed for qPCR analysis for LEDGF mRNA in SRA-hLECs after 5-AzaC exposure for 48 h. Histograms represent the Mean ± S.D. values of three independent experiments. Asterisks indicate a statistically significant difference. Control vs 5-AzaC treated SRA-hLECs, *p < 0.01, **p < 0.001, ***p < 0.0001. **C** 5-AzaC treatment restored the LEDGF transcription inhibited by oxidative stress. Co-transfected SRA-LECs with LEDGF-CAT reporter plasmid (−170/ + 35 nts) or empty CAT vector along with pEGFP vector. Transfectants were exposed to varying doses of H_2_O_2_ in the presence or absence of 5-AzaC (5 μM). After 48 h cell lysate was prepared and CAT activity was measured. Transfection efficiencies were normalized with protein concentration and GFP values (O.D. Ex485/Em530). Values are Mean ± S.D. of three independent experiments and presented in the form of histograms. Control vs H_2_O_2_ treated SRA-hLECs and H_2_O_2_ alone vs H_2_O_2_ + 5-AzaC treated SRA-hLECs, **p < 0.001, ***p < 0.0001. **D** 5-AzaC treatment restored LEDGF mRNA in SRA-LECs facing oxidative stress. Total RNA was isolated from 5-AzaC treated SRA-hLECs facing increasing concentrations of H_2_O_2_ for 120 h. Real-time PCR was conducted for LEDGF mRNA using a specific probe. Histograms represent the Mean ± S.D. values of three independent experiments. Control vs H_2_O_2_ treated SRA-hLECs and H_2_O_2_ alone vs H_2_O_2_ + 5-AzaC treated SRA-hLECs, *p < 0.01, **p < 0.001, ***p < 0.0001. **E** 5-AzaC treatment enhanced the expression of Hsp27 mRNA in SRA-hLECs under increasing oxidative loads. Total RNA was isolated from SRA-hLECs exposed to increasing concentrations of H_2_O_2_ in the presence or absence of 5-AzaC (5 μM) for 120 h. Total RNA was isolated, and qPCR was carried out to examine the levels of Hsp27 mRNA using specific primers (Table 1). Histograms represent the Mean ± S.D. values of three independent experiments. Control vs H_2_O_2_ treated SRA-hLECs and H_2_O_2_ alone vs H_2_O_2_ + 5-AzaC treated SRA-hLECs, *p < 0.01, **p < 0.001, ***p < 0.0001. **F** 5-AzaC treatment reversed the methylation status of the LEDGF promoter. M.S.P. analysis of LEDGF promoter was conducted in SRA-hLECs facing varying doses of H_2_O_2_ in the presence or absence of 5-AzaC (5 μM) as shown. The figure is representative of three independent experiments. **G** 5-AzaC treatment rescued SRA-hLECs against oxidative stress. SRA-hLECs were exposed to varying concentrations of H_2_O_2_ in the presence or absence of 5-AzaC (5 μM). To assess cell viability, MTS assay was conducted after 48 h as described in Materials and Methods. The histogram represents the Mean ± S.D. values of three independent experiments. Control vs H_2_O_2_ treated SRA-hLECs and H_2_O_2_ alone vs H_2_O_2_ + 5-AzaC treated SRA-hLECs, **p < 0.001, ***p < 0.0001.

Next, we asked whether the LEDGF repression can be derepressed by the application of demethylating agents, 5-AzaC. We found that 5-AzaC significantly increased the cell viability (Fig. 7A) with a significant increase of LEDGF expression (Fig. 7B). Next, we transfected the LEDGF promoter (−170/ + 35) containing all three Sp1 sites into SRA-hLECs and exposed the transfectants to H_2_O_2_ as shown in figure in the presence/absence of 5-AzaC. Promoter assay results showed a significant increase in the promoter activity in 5-AzaC treated SRA-hLECs (Fig. 7C). In subsequent experiments, co-treatment of H_2_O_2_ and 5-AzaC could enhance the expression of LEDGF (Fig. 7D) and its target gene Hsp 27 (Fig. 7E). Next, we carried out experiments to determine the possibility of reversing the methylation status of the LEDGF promoter by 5-AzaC. SRA-hLECs were exposed to oxidative stress (Fig. 7F). MSP analysis of genomic DNA showed a change in the methylation status as the band intensity in the methylated PCR product was increased in H_2_O_2_-exposed samples (Fig. 7F), resulting in a reduction of cell viability (Fig. 7G). However, we observed treatment of 5-AzaC, significantly rescued the cells from oxidative damage.

## Discussion

The promoter regions of housekeeping genes are mostly highly GC-rich, and these genes are expressed ubiquitously, such as MAZ or LEDGF [16, 40, 41]. LEDGF has multifunctional activities [13, 15, 16, 23, 28, 29, 34, 42] and that the activities of LEDGF dependent on its regulation and cellular expression in response to the stimuli of the cellular microenvironment [23, 30, 32, 35, 38, 43]. However, no information is available on the epigenetic regulation of LEDGF transcription, and the activity of Sp1 at its binding sites during aging/oxidative stress and disease state (like cataractogenesis). Thus, in this study, we examined the epigenetic regulation of LEDGF gene in human aging and cataractous lenses, including redox-active mLECs or SRA-hLECs. We found that aging and oxidative stress caused aberrant methylation of the CpG island containing all three Sp1 binding sites (Figs. 1 and 2), resulting in the silencing of LEDGF and this event was related to the increased methylation of CpG island (Figs. 3 and 4). We also observed that the CpG island aberrantly methylated in aging, causing LEDGF repression (Figs. 5 and 6). Nonetheless, DNA-methylation of genes leading to the suppression of the genes has been reported in various model systems [3, 41, 44–46]. Paradoxically, promoter-DNA hypermethylation for certain genes has been found to activate gene transcription [2, 45, 47, 48]. The promoters of housekeeping genes are TATA-less and primarily regulated via Sp1/GC sites, like LEDGF [11, 15, 16, 49].

It was intriguing to observe that the CpG island present in the LEDGF promoter encompasses all three functional Sp1/GC sites (Fig. 2C). It is known that the promoters with CpG island are the target for DNA methylation, and the methylation status regulates the expression of genes [50]. Increasing studies have shown that the magnitude of oxidative stress alters methylation/demethylation patterns of genes and their expression [45, 51, 52]. In this context, we think that the methylation of high CpG contents of LEDGF promoter is responsible for its cellular expression. Indeed, our results revealed that the repression of the LEDGF gene was linked to the aberrant DNA methylation of the promoter and disrupted Sp1 binding (Figs. 1, 2, 4 and 6). Furthermore, disrupted Sp1 binding to GC can also be due to the changes in the recruitment of MeCP2 to the methylated CpG dinucleotides of LEDGF promoter during aging/oxidative stress. Similar observations have been reported previously with collagen, collagenase, Myb, Egr-1, and ferritin transcriptions [53–65]. These studies are in agreement with our finding showing that oxidative stress represses LEDGF expression. Also, both MSP and bisulfite sequencing analyses, showed similar results with LEDGF promoter.

A large body of evidence showed [66] that heterochromatin structure changes with advancing age, leading to concomitant gene expression changes [67, 68]. Our results are consistent with the previous reports, showing that the epigenetic changes in chromatin architecture and gene expression are associated with aging and oxidative stress [69, 70]. This could be explained through our ChIP experimentation, which showed the variations in the recruitment of molecules responsible for the methylation process, like DNMTs and MeCP2 to the GC sequences of the promoter (Figs. 5 and 6). We also anticipate that LEDGF may be engaged with chromatin tethering or protein-protein interaction [30, 35, 43], and this phenomenon may affect LEDGF’s cellular abundance. However, this possibility could be ruled out as LEDGF is transcriptionally regulated by Sp1 wherein disruption of Sp1/GCs binding only can interrupt LEDGF transcription or expression. More recently, DNA-induced dimerization of LEDGF has been reported [30, 31, 71]. However, the self-interaction of proteins to form dimers or multimers is naturally destined in the regulation of protein to perform the allotted cellular process in favor of cellular health [72]. Nevertheless, the dimerization of LEDGF may yield a repeating of LEDGF’s structural domain on it that can enable the chromatin/DNA to dock on its surface [73]. However, our data revealed that the reduction of LEDGF transcripts (as well as protein) was in the DNA methylation-dependent during aging/oxidative stress (Fig. 6), suggesting that LEDGF dimerization should not affect its expression. Moreover, the silencing of LEDGF that occurred due to the epigenetic remodeling, we also confirmed by employing 5-AzaC in the experiments, wherein the treatment revived the LEDGF expression and its target gene Hsp 27, (a heat shock protein 27, a cytoprotective protein). Also, our M.S.P. experiments data showed the derepression of LEDGF promoter by 5-AzaC treatment, resulting in increased cell viability (Fig. 7). It has been shown that 5-AzaC treatment reactivates hypermethylated p16 gene promoter [74]. Nevertheless, more insight into this study will be required to understand the molecular mechanisms behind oxidative or age-related pathogenesis, at least in the eye lens.

In sum, our epigenetic reprogramming analysis of the LEDGF gene in response to oxidative stress and aging lenses/LECs led to new insights into the biological changes, showing that the dynamical process, DNA-methylation/demethylation of the promoter could be a cause for LEDGF’s diverse functions. It was intriguing to observe that all three sites of Sp1 binding sequences were differentially methylated in response to aging/oxidative stress/cataractogenesis, demonstrating the importance of the sequence patterns of Sp1 binding sites in LEDGF promoter, which warrants further investigation. Because of the readily reversible feature of DNA methylation, we anticipate that this carries a great potential for the use of selective therapeutics to delay/prevent aging/oxidative stress-related disorders explicitly connected to aberrant DNA methylation.

## Materials and methods

### Cell culture

Two types of lens epithelial cells (LECs) were used: (1) primary hLECs isolated from deceased persons of different ages and (2) a cell line (SRA01/04) immortalized with SV40. To avoid confusion, the remaining text will designate the primary human LECs as hLECs and, the immortalized LECs as SRA-hLECs.

Primary hLECs were isolated from normal eye lenses of deceased persons or healthy donors of different ages (18, 21, 26, 46, 48, 52, 64, 66, 68, 75, 75, 81 years old) obtained from the Lions Eye Bank, Nebraska Medical Center, Omaha, NE, and National Disease Research Interchange (NDRI), Inc., PA, USA. According to regulation HHS45CFR 46.102(f), studies involving material from deceased individuals are not considered human subject research as defined at 45CFR46.102(f) 10(2) and do not require IRB oversight. Due to the limited sample size, eye lenses were divided into groups by age for the experiments. Briefly, lenses were washed with cold PBS, and were directly used to examine expression of LEDGF to avoid cell culture effects. Notably, these lenses were clinically confirmed catatractous and non cataractous lenses.

The SRA-hLECs were generated from 12 infants who underwent surgery for retinopathy of prematurity [75] (a kind gift of late Dr. Venkat N. Reddy, Eye Research Institute, Oakland University, Rochester, MI, U.S.A). These cells were maintained in Dulbecco’s Modified Eagle Medium (DMEM, Invitrogen, Waltham, MA, USA) with 15% fetal bovine serum (FBS, Atlanta Biologicals, Atlanta, GA, USA), 100 µg/ml streptomycin, and 100 µg/ml penicillin in 5% CO_2_ environment at 37 °C as described previously [76]. Cells were harvested and cultured in 96, 24, 48 or 6 well plates and 60- or 100-mm petri dishes according to the requirements of the experiments.

### Mouse lens epithelial cells (mLECs) isolation from lenses of C57BL/6 mouse

All animal experiments followed the recommendations set forth in the “Statement for the Use of Animals in Ophthalmic and Visual Research” by the Association for Research in Vision and Ophthalmology. All studies on animals were approved by the Institutional Animal Care and Use Committee (IACUC), University of Nebraska Medical Center (UNMC), Omaha, NE. Animals were maintained under specific pathogen-free conditions in an animal facility. Nevertheless, we have used *Prdx6*^*−/−*^ mutant mice which are maintained on fully inbred C57BL/6 background [27], and wild-type mice of the same sex and age (*Prdx6*^*+/+*^). This minimizes the variation due to genetic background. LECs isolated from Prdx6-targeted mutants (*Prdx6*^*−/−*^) and wild-type (*Prdx6*^+/+^) mice were generated and maintained in Dulbecco’s Modified Eagle’s Medium (DMEM; Invitrogen, Carlsbad, CA, USA) with 10% FBS (Atlanta Biologicals, Inc., Flowery Branch, GA, USA) as described earlier [27]. Western blot analysis was carried out to confirm the presence of αA-crystalline [27], a specific marker of LECs. Cells from 3–5 passages were used for the experiments in this study.

### Quantitative real-time PCR

Total RNA was isolated from the different age groups of human lenses/hLECs, SRA-hLECs exposed with different concentrations of H_2_O_2_ or 5-AzaC or H_2_O_2_ + 5-AzaC in serum-free DMEM as indicated in Figure legends, and *Prdx6*^*+/+*^ and *Prdx6*^*−/−*^ mLECs using Trizol reagent (Invitrogen, USA). Following integrity validation, RNA was converted to cDNA using Superscript II RNAase H-Reverse Transcriptase as described previously [16]. Quantitative real-time PCR was performed using LightCycler® 480II (Roche Diagnostic Corporation, Indianapolis, IN, USA) as described earlier [11, 15, 16, 24]. The comparative Cp method was used to calculate relative fold expression levels using Lightcycler 480 software, release 1.5.0 SP3. The Cps of target genes was normalized to β-actin as an endogenous control in each group. PCR conditions consisted of 10 min hot start at 95 °C, followed by 45 cycles of 10 s at 95 °C, 30 s at 60 °C, and 10 s at 72 °C. Primer sequence as shown in Table 1.

**Table 1:** RT-qPCR primer sequence.

Gene Name	Forward Primer	Reverse Primer
hLEDGF	5′-CAGCAACAGCATCTGTTAATCTAAA-3′	5′-GGGCTGTTTTACCATTTT GG-3'
hHsp27	5′-TCCCTGGATGTCAACCACTT-3′	5′-GATGTAGCCATGCTCGTCCT-3′
hβ-actin	5′-CCAACCGCGAGAAGATGA-3′	5′-CCAGAGGCGTACAGGGATAG-3′
mLEDGF	5′-AGCAAGTCCTAAGAGAGGACGA-3′	5′-GGACAAGGCTGCTTTACCAC-3′
mPrdx6	5′-TTTCAATAGACAGTGTTGAGGATCA-3′	5′-CGTGGGTGTTTCACCATTG-3′
mβ-actin	5′-CTAAGGCCAACCGTGAAAAG-3′	5′-ACCAGAGGCATACAGGGACA-3′

### Protein isolation and Western blot analysis

Total cell lysate [27], and Nuclear and cytosolic extraction were prepared from LECs as described earlier [11]. Equal amounts of total cell lysate and nuclear extract protein samples were resolved onto a 4–20% SDS-PAGE, blotted onto PVDF membrane (Perkin Elmer), and immune stained with primary antibodies at the appropriate dilutions of LEDGF monoclonal antibody (Cat. no. 611715, B.D. Biosciences,). DNMT1 (Cat. no. 5032), DNMT3a (Cat. no. 2160), DNMT3b (Cat. no. 2161), and Histone H3 (Cat. no. 4499) antibodies were obtained from Cell Signaling Technologies (Danvers, MA, USA). Prdx6 (Cat. No. ab16947), Sp1 (Cat. No. ab77441), MeCP2 (Cat. No. ab2828) and HDAC1 (Cat. no. ab46985) antibodies were purchased from Abcam (Cambridge, United Kingdom). Membranes were incubated with horseradish peroxidase-conjugated secondary antibodies (1:1500). Specific protein bands were visualized by incubating the membrane with luminol reagent (Santa Cruz Biotechnology, Cat. no. sc-2048) and recorded with FUJIFILM-LAS-4000 luminescent image analyzer (FUJIFILM Medical System Inc.). To ascertain comparative expression and equal loading of the protein samples, the membrane stained earlier was stripped and re-probed with β-actin antibody (Cat. no. ab25894, Abcam).

### Small interfering RNA (siRNA) assay

The LEDGF-specific small interfering (si) RNA expression plasmid was designed according to the method described earlier [12, 15, 77]. The sequence was selected from location 1340–1360 (5′-AAA GAC AGC ATG AGG AAG CGA-3′). The sense and antisense oligonucleotides with the internal loop were synthesized by Invitrogen. These were annealed and ligated into the *BamHI* and *HindIII* sites of pSilencer 4.1-CMV hygro (Ambion, Cat. no. AM5777) pSilencer 4.1- pCMVhygro expressing a scrambled siRNA (Ambion, Cat. no. AM5777) was used as a control. siRNA constructs were transfected into SRA-hLECs with Neon transfection systems (Invitrogen). 24 h of post-transfection, stable transfected cells were selected using 400 μg/ml of hygromycin (Invitrogen, Cat. no. 10687-010) for 9 days. Silencing was confirmed by LEDGF protein expression.

### Cell survival assay (MTS assay)

To examine the effect of H_2_O_2_-induced oxidative stress on cell viability, LEDGF over- and under-expressed SRA-hLECs were harvested and cultured in 96 or 48 well plates. After 24 h, these cells were exposed to different concentrations of H_2_O_2_ in serum-free DMEM as indicated in figure legends, and then subjected to MTS assay. Similarly, SRA-hLECs were harvested and cultured in 96 or 48 well plates. After 24 h, these cells were exposed to different concentrations of H_2_O_2_ alone or H_2_O_2_ + 5-AzaC in serum-free DMEM and then subjected to MTS assay. A colorimetric MTS assay (Promega, Cat. no. G3582) was performed as described earlier [15, 78]. This assay of cellular proliferation uses 3-(4,5-dimethylthiazol-2-yl)-5-(3-carboxymethoxyphenyl)-2 to 4-sulfophenyl-2H-tetrazolium salt. Upon being added to a medium containing viable cells, MTS is reduced to a water-soluble formazan salt. The O.D. 490 nm values were measured after 4 h with an ELISA reader (DTX 880 Multimode Detector, Molecular Device).

### Assay for intracellular redox state

Intracellular redox state levels were measured using the fluorescent dye, H_2_-DCF-DA as described earlier [15, 78]. SRA-hLECs (1 × 10^4^) were cultured in 96-well plates and were subjected to different concentrations of H_2_O_2_ (50, 70, 90, and 100 µM) in serum-free DMEM media. *Prdx6*^*+/+*^ and *Prdx6*^*−/−*^mLECs were cultured for 24 h in 96 well plates and then subjected to assess the ROS level. Cells were washed once with HBSS and incubated in the same buffer containing 10 μM of H_2_-DCF-DA. This is a nonpolar compound that is converted into a polar derivative (dichlorofluorescein) by cellular esterase following incorporation into cells. Following 30 min of incubation at room temperature, intracellular fluorescence was detected with Ex485/Em530 nm using Spectra Max Gemini EM (DTX 880 Multimode Detector, Molecular Device).

### Preparation of LEDGF promoter-CAT construct

The genomic human phagemid P1 clone (Genomic System) was used to construct 5′ flanking region of the human LEDGF gene as reported previously [11, 12, 15, 16]. The genomic P1 clone comprising the LEDGF gene was subjected to Genomic PCR with primers containing *Mlu* I and *Nhe* I, and fragments encompassing −315 to +35, −170 to +35, and −63 to +35 bps were ligated into basic pCAT vector (Promega, Cat. no. E1871) with the appropriate restriction enzymes as reported earlier [16]. The plasmid was amplified by transforming into bacteria (Top 10, Invitrogen, Cat. no. C4040-06) using the standard method, sequenced, and used for CAT assay.

### Transfection and chloramphenicol acetyltransferase assay (CAT assay)

CAT assay was performed using a CAT-ELISA (Roche Diagnostics) kit as described earlier [11, 16]. Briefly, SRA-hLECs were cultured at a density of 5 × 10^5^ cells in 5 ml of DMEM containing 15% FBS per 60-mm Petri dish in a 37 °C incubator containing 5% CO_2_. Cells were washed with the same medium and transfected/cotransfected with a Neon transfection system (Invitrogen) with promoter/CAT reporter constructs along with 1 μg of pEGFP vector. After 24 h of incubation, cells were subjected to H_2_O_2_ alone or H_2_O_2_ + 5-AzaC exposure in serum-free DMEM, and an extract was prepared. Protein concentration was normalized, and CAT-ELISA was performed to monitor CAT activity. The absorbance was measured at 405 nm using a microtiter plate ELISA reader. The concentration of plasmid DNA was equal in each transfection to maintain a similar DNA burden on cells and to avoid any nonspecific effect(s). Transactivation activities were adjusted for transfection efficiencies using GFP values (O.D. at Ex485/Em530).

### Chromatin immunoprecipitation (ChIP) analysis

SRA-hLECs were exposed to different concentrations of H_2_O_2_ in serum-free DMEM medium and incubated up to 120 h as indicated in Figure-legends. ChIP analysis was conducted by the ChIP-IT Express kit (Active Motif, Cat. no. 53008) according to the manufacturer’s protocol and as described earlier [11, 15, 16]. ChIP grade specific anti-Sp1 (ab13370), anti-5-methyl Cytosine (ab10805), anti-MeCP2 (ab195393), anti-HDAC1 (ab46985), and Control IgG (ab171870) antibodies were used. Primers used in ChIP analyses: Unrelated to Sp1 site primers: Forward, 5′-CAACGTGGCAAAACCCTATC-3′ and reverse, 5′-CCTGACCTCAAGTGGACCAT-3′; Sp1 sites: Forward, 5′-GCCACTTTCTCCCTA ACACG-3′ and reverse, 5′-AACCCTACGTCCCCAAGTTC-3′. The thermocycler programs were as follows: 94 °C for 3 min; 35 cycles of 94 °C for 20 sec, 55 °C for 30 s, 72 °C for 30 s; and extended at 72 °C for 3 min at the end. ChIP assays were conducted via standard PCR amplification (Go-Taq, Promega, Cat. no. M8291) followed by agarose gel electrophoresis. Amplified DNA bands were resolved on 2.5% agarose gels, and images were obtained using a FUJIFILM-LAS-4000 luminescent image analyzer.

### In vitro methylation experiment

An aliquot of 4 µg plasmid DNA (pLEDGF-CAT) was incubated with M.SssI methyltransferase (New England Biolabs) 4 h at 37 °C in a reaction mixture containing 10 mM Tris-HCl, pH 7.9, 50 mM NaCl, 10 mM MgCl2, 640 µM *S*-adenosylmethionine (SAM), 10 mM EDTA and 1 mM dithiothreitol (DTT). The reaction was terminated by heating at 65 °C for 20 min followed by phenol extraction and chloroform/isoamyl alcohol (24:1). The DNA was precipitated with ethanol. The efficiency of methylation was controlled by the digestion of methylated pLEDGF-CAT with BstUI. Prior to transfection, the methylated DNA was purified by agarose gel electrophoresis.

### Methylation-specific PCR (M.S.P.) and Bisulfite sequencing

Genomic DNA was extracted from cells or cells exposed multiple times with variable doses of H_2_O_2_ treatment for 120 h or from human lenses/LECs of variable ages as mentioned elsewhere by using the commercially available kit (genomicPrep Mini Spin kit, G.E. Healthcare, Cat. no. 28-9042-75), and DNA was then treated with bisulfate as described in Methyl Detector TM, Instruction Manual (Active Motif, Cat. no.55001) as well as described earlier [11, 79]. Briefly, a conversion reaction was performed in a PCR tube containing 1 μg denatured genomic and conversion reaction buffer with hydroquinone and 3 M sodium bisulfite in total volume of 260 μl. Tubes were placed in a thermocycler set at 94 °C for 3 min, then at 50 °C conversion for 5 h, followed by a hold (4 °C). The reaction mixture containing the bisulfite-modified DNA was purified with a DNA purification column (Active Motif, Cat. no. 55001). The eluted DNA was analyzed by M.S.P. using region-specific primers chemically synthesized using a designer program at The MethPrimer website; www.urogene.org/methprimer. Primers and PCR conditions for LEDGF promoter were as follows: unmethylated forward primer, 5′-GAG GTT TGG ATA TTT GTT TTT AAA ATT-3′; reverse primer, 5′-CCA CCC CTA CTA ACT TCT CTA CAT A-3′, and methylated forward primer, 5′-GTT CGG ATA TTC GTT TTT AAA ATC-3′; reverse primer, 5′-GCC CTA CTA ACT TCT CTA CGT A-3′. The thermocycler programs were as follows: 94 °C for 3 min; 30 cycles of 94 °C for 30 s, 50 °C for 30 s, 72 °C for 30 s; and extended at 72 °C for 10 min at the end. The reaction products were analyzed onto a 2.25% low molecular weight agarose gel. In experiments included with 5-AzaC, the treatments were done simultaneously and continued up to 120 h along with H_2_O_2_ treatment. For detecting the critical CpG that is methylated, the PCR amplified products from bisulfite converted DNA were cloned into TOPO TA vector using standard cloning protocol and subjected the cloned plasmid to next-generation sequencing.

### Statistical analysis

All the statistical analyses were done in GraphPad Prism software. Comparison between the two groups was done with Student’s *t*-test. Multiple comparisons were done by ANOVA. A *p* < 0.01, <0.001, and <0.0001 was defined as indicating a statistically significant difference.

### Supplementary information

Supplementary Material

## Data Availability

Data are contain within the article.
