# Supplementary figures and images for: DNA methylation as an epigenetic mechanism in the regulation of LEDGF expression and biological response in aging and oxidative stress
Source: Cell Death Discov. 2024 Jun 22;10:296. doi: 10.1038/s41420-024-02076-2 (PMC11193803; doi:10.1038/s41420-024-02076-2)

Figure 1C

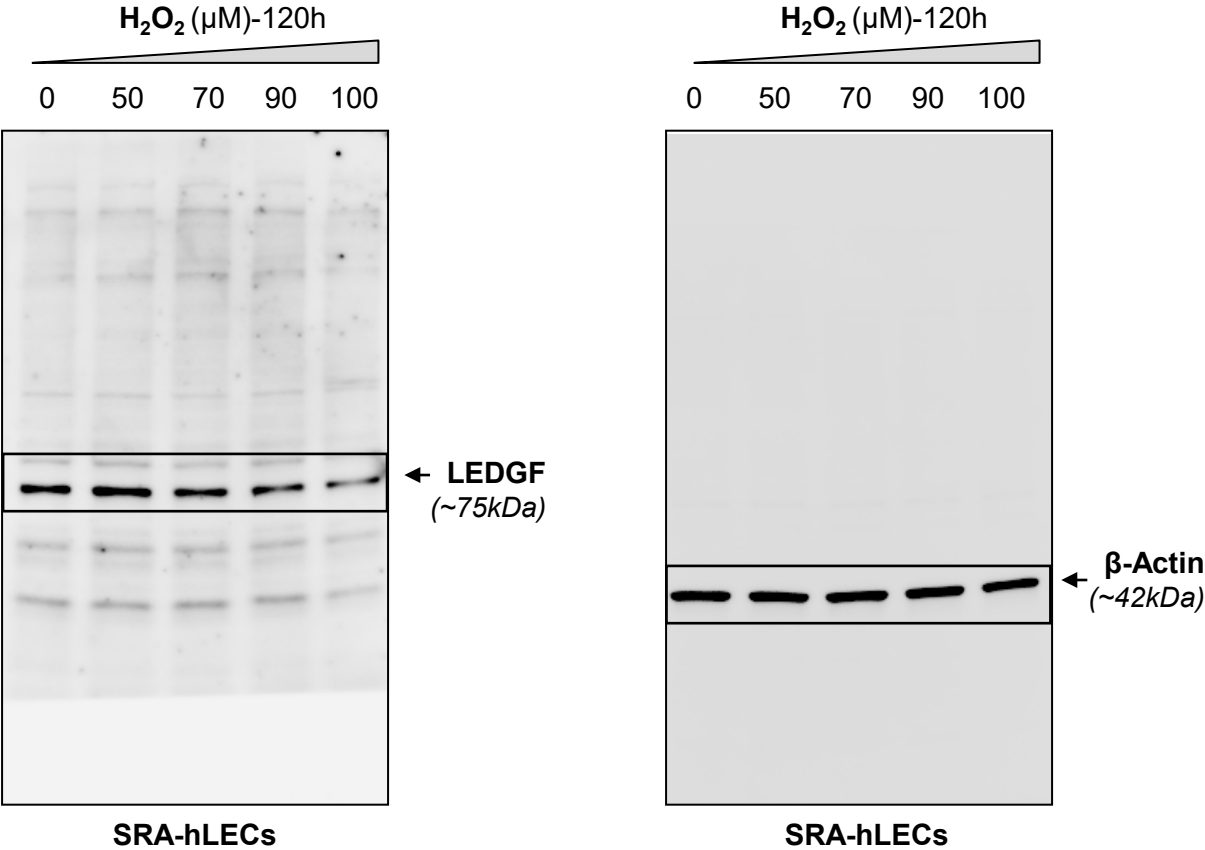

Figure 1E

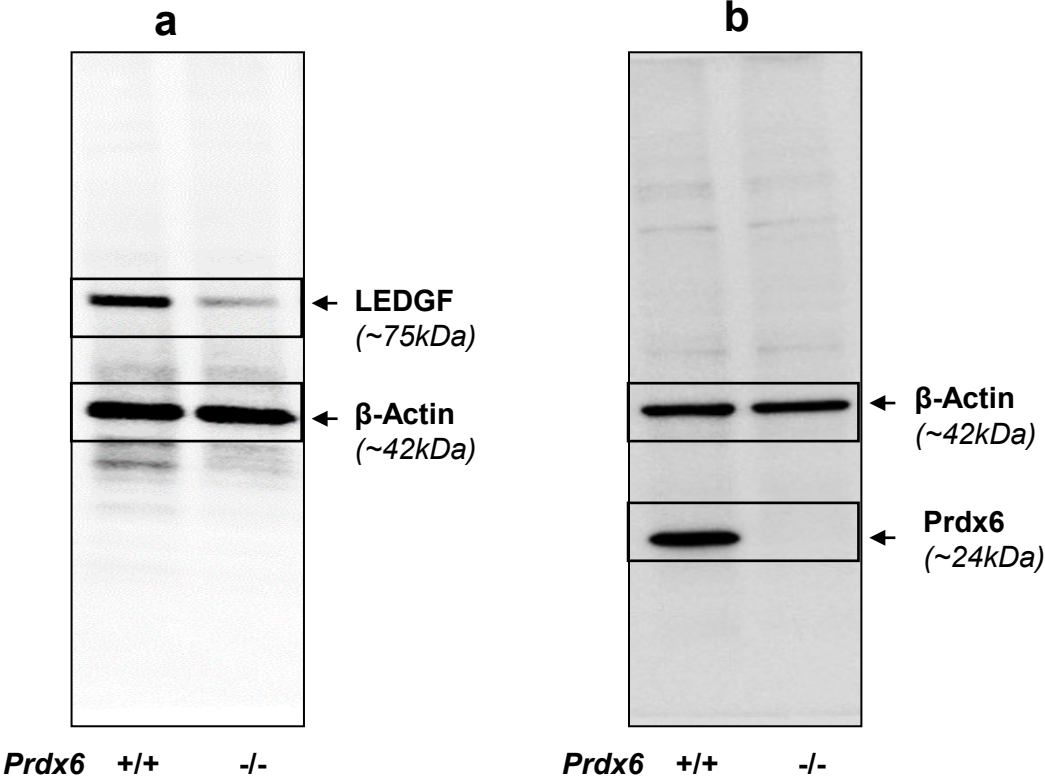

Figure 3A

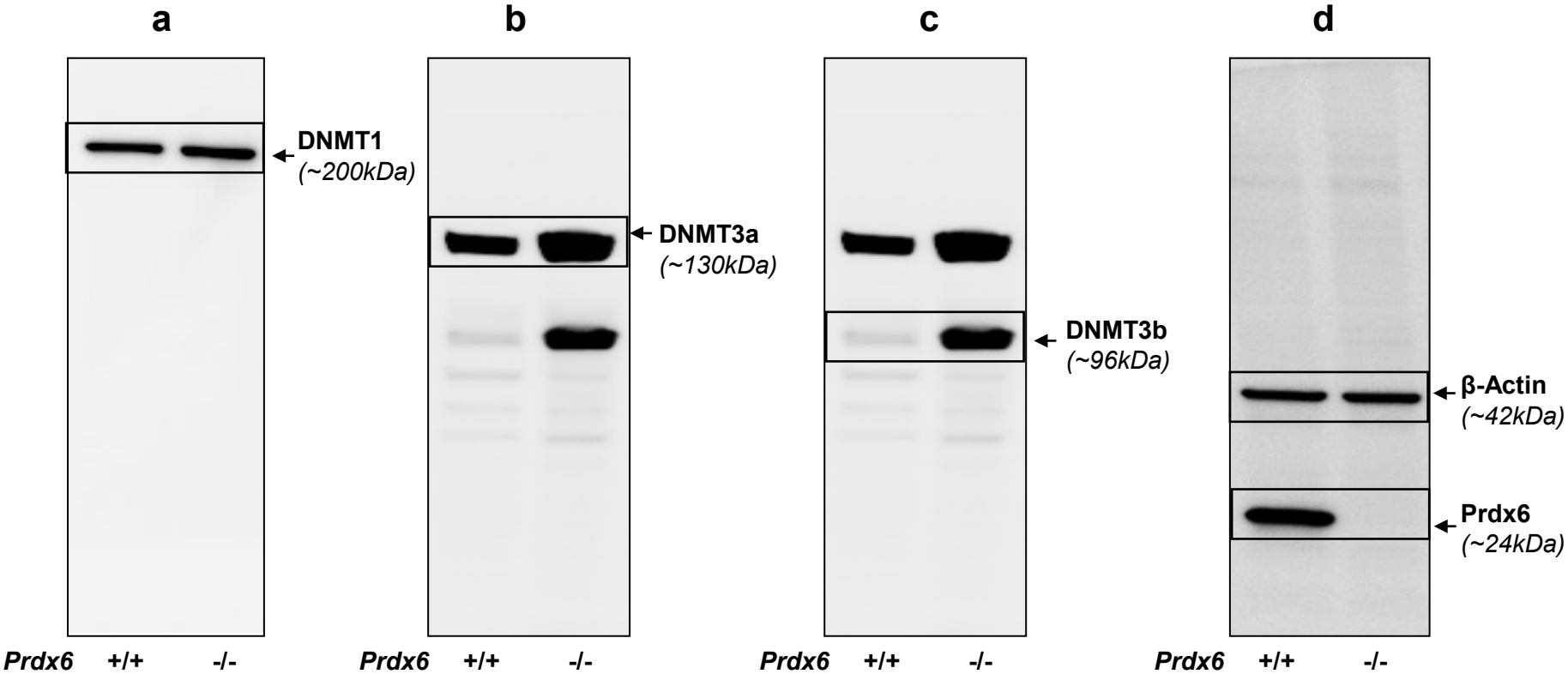

Figure 3B

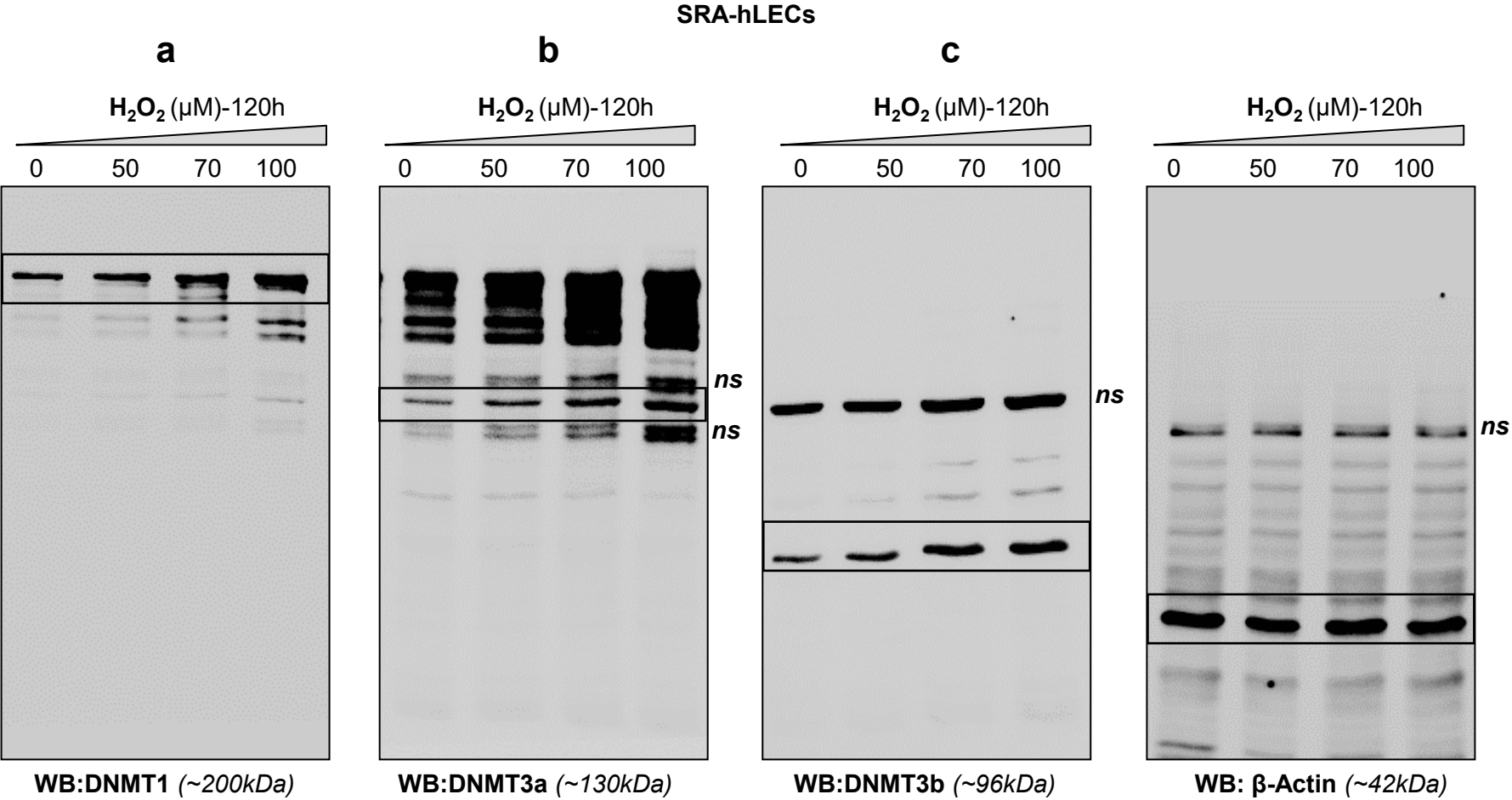

Figure 5A

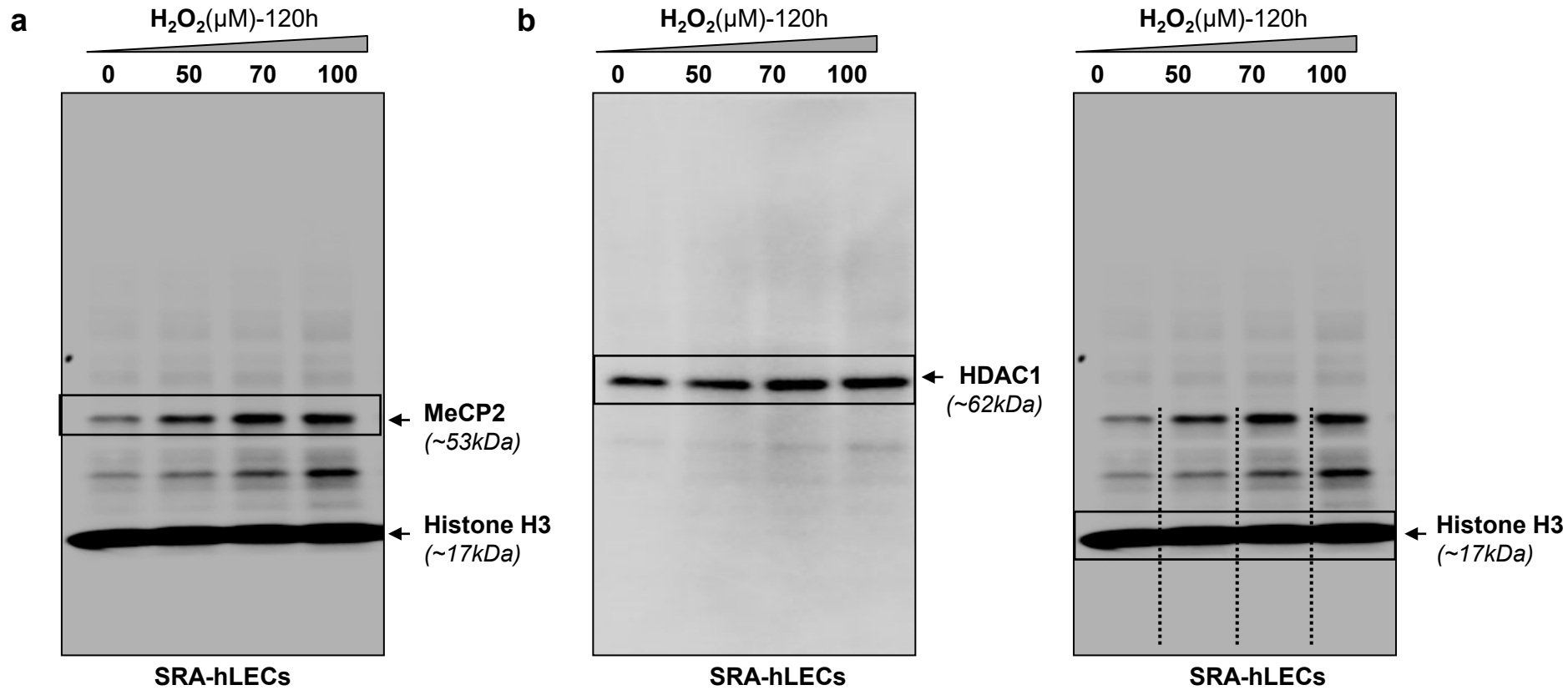

Supplement: Supplementary file 1 — Supplementary Material [file 41420_2024_2076_MOESM1_ESM.pdf]
